# Elongation Factor-G (*fusA*) Mutations That Confer Fusidic Acid Resistance in *Staphylococcus haemolyticus*

**DOI:** 10.3390/antibiotics15060589

**Published:** 2026-06-09

**Authors:** Cheng-Mao Ho, Lee-Chung Lin, Yu-Hsiang Ou, Kai-Hsiang Lin, Jang-Jih Lu

**Affiliations:** 1 Division of Clinical Pathology, Taichung Tzu Chi Hospital, Buddhist Tzu Chi Medical Foundation, Taichung 427213, Taiwan; chengmao.ho@tzuchi.com.tw; 2 Division of Laboratory Medicine, Taichung Tzu Chi Hospital, Buddhist Tzu Chi Medical Foundation, Taichung 427213, Taiwan; tc102230@tzuchi.com.tw; 3 Department of Laboratory Medicine and Biotechnology, College of Medicine, Tzu Chi University, Hualien 970374, Taiwan; 4 Department of Pathology, School of Medicine, Tzu Chi University, Hualien 970374, Taiwan; 5 Department of Laboratory Medicine, Linkou Chang Gung Memorial Hospital, Taoyuan 333423, Taiwan; leollc@gmail.com (L.-C.L.); planet7668@cgmh.org.tw (Y.-H.O.); 6 Department of Life Sciences, National Chung Hsing University, Taichung 40227, Taiwan; 7 Division of Clinical Pathology, Taipei Tzu Chi Hospital, Buddhist Tzu Chi Medical Foundation, New Taipei 231405, Taiwan

**Keywords:** *Staphylococcus haemolyticus*, fusidic acid resistance, *fusA*, elongation factor G, antibiotic resistance, fitness cost

## Abstract

**Background/Objectives:** *Staphylococcus haemolyticus* is a common skin commensal that has emerged as a multidrug-resistant nosocomial pathogen, with sequence type 42 frequently implicated in clinical settings. The genetic basis of fusidic acid (FA) resistance mediated by mutations in elongation factor-G (*fusA*/EF-G) has not been systematically characterized in *S. haemolyticus*. **Methods:** Five representative FA-susceptible *S. haemolyticus* isolates were selected. In vitro FA resistance was induced by incubating each isolate on Mueller–Hinton agar containing 4 µg/mL FA at 37 °C for 48 h. Resistant colonies were recovered and *fusA* was sequenced by Sanger sequencing to identify mutations. Growth doubling times of EF-G mutant isolates were measured and compared with those of the parental susceptible strains. Potential fitness-compensatory changes in *fusA* were assessed by serial passage of selected mutants for ten successive passages and re-sequencing of *fusA*. **Results:** A total of 28 FA-resistant colonies were recovered. Sequencing identified mutations at seven nucleotide loci corresponding to ten distinct amino acid substitutions in EF-G: Q115L, L430S, E433G, G452C, H457Y, H457N, H457L, H457Q, R464L, and A655G. The mean doubling time of EF-G mutant isolates was significantly longer than that of the wild-type parental strains (mutants: 55.75 ± 7.78 min, n = 28; wild type: 40.78 ± 4.13 min, n = 5; Welch’s two-sample t test: t = −6.34, df ≈ 10.0, two-tailed *p* < 0.0001). Following ten serial passages, we did not detect compensatory mutations in *fusA* that restored the ancestral growth rate. **Conclusions:** FA resistance in *S. haemolyticus* can be rapidly induced in vitro through mutations in EF-G/*fusA*. Compared with previously reported EF-G mutations in other staphylococci, we identified two novel substitution sites (L430S, E433G) and previously unreported substitutions at established resistance positions (H457N, H457L, A655G). Mutations in EF-G (encoded by *fusA*) were associated with a measurable in vitro fitness cost; following ten serial passages, compensatory mutation was not detected in *fusA*. These findings support systematic surveillance of EF-G/*fusA* mutations in clinical *S. haemolyticus* isolates. Further studies should address their prevalence, stability, transmissibility, and clinical impact, particularly where FA use is increasing and patient populations are vulnerable.

## 1. Introduction

Fusidic acid (FA), a steroidal antibiotic structurally related to cephalosporin P1, was originally isolated from the fungus *Fusidium coccineum* and introduced into clinical practice in the 1960s [1]. Although fusidic acid exhibits limited in vitro activity against Gram-negative bacteria such as *Neisseria* spp. and *Moraxella catarrhalis*, its primary antimicrobial activity is directed against Gram-positive organisms, particularly staphylococci. This includes both methicillin-susceptible and methicillin-resistant *Staphylococcus aureus* (MSSA and MRSA), as well as coagulase-negative staphylococcal species [2,3]. The clinical breakpoints for FA are not provided by the Clinical and Laboratory Standards Institute (CLSI) [4], but the European Committee on Antimicrobial Susceptibility Testing (EUCAST) currently defines a susceptible breakpoint of 1 µg/mL under standard dosing regimens (0.5 g orally or intravenously twice daily) [5]. The antimicrobial mechanism of fusidic acid involves binding to elongation factor G (EF-G) during the translocation phase, thereby preventing EF-G release from the ribosome and halting protein synthesis [6].

FA resistance in staphylococci arises through multiple mechanisms. Mutations in the EF-G-encoding gene (*fusA*) can confer high-level resistance (e.g., minimal inhibitory concentration (MIC) > 64 µg/mL) [1]. In contrast, acquisition of resistance genes such as *fusB*, *fusC*, *fusD*, and *fusF*—which encode small proteins that interfere with fusidic acid binding to EF-G—typically results in low-level resistance [1,7,8]. Additionally, mutations in the ribosomal protein L6 gene (*rplF*), designated *fusE*, have been associated with low-level resistance in small-colony variants of *S. aureus* [9]. The prevalence of fusidic acid resistance and its determinants varies across time, geographic regions, and staphylococcal species [10,11,12]. Globally, resistance rates between 2000 and 2020 were reported as 6.7% in MRSA and 2.6% in MSSA [10]. For coagulase-negative staphylococci, resistance rates ranged from 7.2% to 38.6% depending on species and location [12,13]. In clinical isolates, *fusB* and *fusC* are frequently observed and can disseminate horizontally via mobile genetic elements, with *fusA* mutations occurring less commonly but conferring higher-level resistance when present [1,11,12,13,14].

Because *fusA* encodes an essential translation factor, resistance-conferring substitutions often impose a growth or fitness cost [1,15]. Compensatory mutations that restore fitness have been documented in *S. aureus*, and reported single-step mutation rates vary with the magnitude of MIC increase [15,16]. The relatively low genetic barrier to FA resistance in *S. aureus* has prompted recommendations for combination therapy (notably FA plus rifampicin) to reduce the risk of resistance emergence [17]. In vitro studies have demonstrated partial synergistic or additive interactions between these agents [16,17].

*Staphylococcus haemolyticus* is a common human and animal commensal and is generally considered less virulent than *S. aureus* [18]. Nevertheless, particular lineages, most notably sequence type 42, have emerged as multidrug-resistant nosocomial pathogens in several regions, including Taiwan and China [13,19,20,21,22]. During investigations of these strains, novel colonies were observed when fusidic acid-susceptible *S. haemolyticus* isolates were incubated on fusidic acid-containing agar for extended periods. Given the low genetic barrier to fusidic acid resistance in *S. aureus* [15,16], this observation is noteworthy. However, to our knowledge no studies have systematically characterized fusidic acid resistance mechanisms of *fusA* in *S. haemolyticus*. Because the genetic determinants of *fusA-*/EF-G-mediated FA resistance remain uncharacterized in this species, we undertook a systematic characterization of in vitro-selected FA resistance. In this study, we therefore aimed to (1) identify *fusA*/EF-G mutation loci associated with FA resistance in *S. haemolyticus*, (2) quantify the fitness impact of these mutations in vitro, and (3) determine whether compensatory mutations that restore growth rate arise in *fusA* during serial passage.

## 2. Results

### 2.1. Comparison of EF-G (FusA) Between S. aureus and S. haemolyticus

Based on GenBank entries, the EF-G (FusA) sequences of *S. aureus* (GenBank accession AJ237696.1) and *S. haemolyticus* (strain VB19458; GenBank CP045187.2) are identical in length, each comprising 693 amino acids and 2082 nucleotides, including the terminal stop codon TAA (Figures S1-1 and S1-2). Compared with *S. haemolyticus* ATCC 29970 (GenBank CP035291.1), VB19458 carries two synonymous nucleotide changes at codons 176 (ATC → ATT) and 342 (AAG → AAA) that do not alter the encoded amino acids. Pairwise alignment revealed 28 amino acid differences between the two species (Table 1). The longest contiguous region of identity spanned residues 348 to 487. The EF-G sequences from five selected FA-susceptible *S. haemolyticus* isolates matched strain VB19458 (GenBank CP045187.2).

This interspecies comparison was performed to align the two sequences in a common residue-numbering frame, so that the substitutions detected in *S. haemolyticus* could be mapped directly onto the known FA-resistance positions established in *S. aureus* and classified as conserved or novel. Notably, none of the 28 differences coincide with reported *S. aureus* resistance positions, and the identical region (residues 348–487) encompasses domain III, where most resistance loci—including the dominant residue-457 hotspot identified here—reside. The *S. aureus* reference AJ237696.1 was selected because it is the canonical FA-susceptible *fusA* reference used throughout the *S. aureus* resistance literature, ensuring directly comparable residue numbering; the complete, well-annotated ST3 genome VB19458 (CP045187.2) was used as the *S. haemolyticus* reference to ensure full-length, unambiguous *fusA* coverage.

### 2.2. Mutation Analysis of In Vitro-Induced FA-Resistant Strains

After 48 h of incubation of five FA-susceptible parental strains (SH29, SH43, SH53, SH54, SH141) on Mueller–Hinton agar containing 4 µg/mL FA, 28 resistant colonies were recovered (Table 2 and Table 3). Most resistant isolates originated from SH53 (n = 13), followed by SH54 (n = 10), SH43 (n = 2), SH141 (n = 2), and SH29 (n = 1). We identified seven mutation loci and ten distinct amino acid substitutions in EF-G (FusA) that were associated with FA resistance (Table 2; Figures S1-1 and S1-2). Among the 28 in vitro-derived resistant isolates, the most frequent alteration occurred at residue 457, where histidine was replaced in 18 isolates. The predominant substitution was H457Y (C1369T; CAC → TAC), observed in 14 isolates, which increased FA MICs to 64–128 µg/mL. Three additional substitutions at the same residue were detected: H457N (n = 1; C1369A; CAC → AAC), H457L (n = 1; A1370T; CAC → CTC), and H457Q (n = 2; C1371A; CAC → CAA). These variants increased FA MICs to values ranging from 16 µg/mL to >256 µg/mL. Other EF-G (FusA) substitutions linked to FA resistance included Q115L (n = 3; A344T; CAA → CTA), L430S (n = 1; T1289C; TTA → TCA), E433G (n = 1; A1298G; GAA → GGA), G452C (n = 2; G1354T; GGT → TGT), R464L (n = 2; G1391T; CGT → CTT), and A655G (n = 1; C1964G; GCA → GGA). In addition, three synonymous *fusA* mutations were observed: C528T (ATC → ATT), T1158C (GGT → GGC), and T1353A (GGT → GGA). Two synonymous changes (C528T and T1158C) co-occurred with H457Y in ten isolates, while T1353A co-occurred with G452C in one isolate. All *fusA* mutations remained stable after serial propagation to the tenth generation, with no reversion or additional compensatory changes in *fusA* detected.

### 2.3. Doubling Time of EF-G (FusA) Mutant Isolates Compared with Wild-Type Strains

The doubling times of wild-type strains ranged from 34.0 to 44.7 min, whereas those of EF-G (FusA) mutant isolates ranged from 41.7 to 65.9 min (Table 3). The mean doubling time of wild-type isolates was 40.78 ± 4.13 min (n = 5), compared with 55.75 ± 7.78 min (n = 28) for EF-G (FusA) mutants, yielding a mean difference of 14.97 min. Welch’s two-sample *t*-test indicated a statistically significant difference (*t* = −6.34, df ≈ 10.0, two-tailed *p* < 0.0001). The 95% confidence interval for the mean difference was 9.71–20.23 min. The effect size, quantified by Cohen’s *d*, was 2.02, consistent with a very large effect. The nonparametric Mann–Whitney *U* test produced similar results (*U* = 6.5, two-tailed *p* ≈ 0.0015), corroborating the parametric findings. Together, these analyses demonstrate that EF-G (FusA) mutant isolates exhibit significantly prolonged doubling times relative to wild-type strains.

## 3. Discussion

The escalating clinical use of antibiotics has facilitated the global dissemination of multidrug-resistant (MDR) bacteria, including both Gram-positive and Gram-negative species, thereby creating an urgent clinical challenge [23]. Fusidic acid (FA), which has a unique mechanism of action that limits cross-resistance with other antibiotic classes, is available in intravenous, oral, and topical formulations. Given the limited therapeutic options for MDR Gram-positive infections, the availability of FA, particularly oral formulations for treating methicillin-resistant *Staphylococcus aureus* (MRSA), may promote increased clinical use [1]. The emergence of FA resistance during systemic and topical therapy has been documented [24,25]. Because FA has a relatively low genetic barrier to resistance, an increasing prevalence of FA-resistant staphylococcal isolates is a plausible concern [1,16]. Accordingly, enhanced surveillance and rigorous antimicrobial stewardship are warranted.

Most studies of *fusA*-associated fusidic acid (FA) resistance in staphylococci have concentrated on *S. aureus*, with comparatively few investigations of *S. epidermidis* and *S. pseudintermedius* [26,27,28,29]. Here, we provide the first experimental evidence that *fusA* mutations can confer FA resistance in *S. haemolyticus*, an organism of increasing clinical importance. Structural and functional studies of EF-G (FusA) in *S. aureus* indicate that the protein comprises five domains and that resistance-conferring substitutions can be grouped by their effects on FA binding, EF-G–ribosome interactions, EF-G conformation, or EF-G stability [11,28]. Sequence comparison of EF-G (FusA) between *S. aureus* and *S. haemolyticus* revealed 28 amino acid differences (Table 1). Notably, none of these differences overlapped with the amino acid substitution sites previously reported to confer FA resistance in *S. aureus* (positions 88, 114, 115, 385, 404, 406, 407, 434, 436, 438, 444, 451, 452, 453, 456, 457, 461, 464, 478, 617, 628, 651, 655, 659, 660, 664, 666) [11,27,28]. More than half of these FA-resistance-associated mutations (404, 406, 407, 434, 436, 438, 444, 451, 452, 453, 456, 457, 461, 464, 478) are located in domain III, within regions identical between *S. aureus* and *S. haemolyticus*. Additional FA-associated loci are distributed across domains I, II, and V, but not domain IV, consistent with the structural location of FA binding sites on the ribosome [11]. Position 461, at which L461K has been reported in clinical *S. epidermidis* [26], is conserved between the two species (it is absent from the 28 interspecies differences in Table 1) and lies within this identical domain III region; L461K is therefore an acquired resistance substitution rather than a baseline interspecies difference, and residue 461 remains a plausible—though here unselected—resistance site in *S. haemolyticus*.

Current evidence indicates that *fusA*, *fusB*, *fusC*, *fusD*, *fusE*, and *fusF* mediate FA resistance [1,8]. The genes *fusB*, *fusC*, *fusD*, and *fusF* are generally acquired, while *fusA* and *fusE* arise through spontaneous mutation [1]. Because *fusE* typically confers only low-level resistance and is rare, substantial increases in FA MICs (from ≤1 µg/mL to ≥16 µg/mL) are most often attributable to mutations in *fusA* in this study. In our in vitro selection experiments, we identified seven mutated loci and ten distinct amino acid substitutions in EF-G (FusA) that were associated with elevated FA MICs in *S. haemolyticus* (Table 2). Two substitutions, L430S and E433G, have not been reported previously in other staphylococci and increased FA MICs to 16 µg/mL. At residue 457, we observed four different substitutions, H457Y and H457Q, which have been reported previously in other staphylococci [15,27,30], and two novel variants, H457N and H457L, which conferred high-level resistance (MIC ≥ 28 µg/mL). At locus 655, we observed A655G, associated with FA resistance (MIC = 32 µg/mL), whereas prior reports described A655E in *S. aureus* [9]. These findings indicate both convergence on known resistance hotspots in other staphylococci and the emergence of novel substitutions that can mediate FA resistance in *S. haemolyticus*.

To interpret these substitutions mechanistically, we mapped them onto the EF-G (FusA) structure of *S. aureus*, which is directly applicable because every residue mutated here (115, 430, 433, 452, 457, 464, and 655) is conserved between the two species (none appears among the 28 interspecies differences in Table 1). EF-G comprises five domains, and fusidic acid binds at the interface of domains I, II, and III, trapping EF-G on the ribosome after GTP hydrolysis and translocation [6,28]. Most of our substitutions cluster in domain III, the principal FA-resistance region: L430S and E433G lie immediately adjacent to the established resistance cluster (residues 434, 436, 438), whereas G452C, the residue-457 variants, and R464L fall within it. Residue 457 lines the fusidic acid-binding pocket, consistent with it being the dominant hotspot; the strong dependence of MICs on side-chain chemistry at this position (H457Y/N/L conferring MIC ≥ 64 µg/mL, up to >256 µg/mL, versus H457Q at 16 µg/mL) is consistent with a direct role at the drug interface. L430S and E433G, being peripheral to the pocket, more plausibly perturb domain III positioning than contact the drug directly, in line with their moderate MICs (16 µg/mL). Q115L maps to the G domain (domain I) near the GTPase/switch region, and A655G maps to domain V, which mediates EF-G–ribosome contacts; both more plausibly act on EF-G conformational dynamics and ribosome interaction than on direct drug binding (cf. the previously reported A655E [9]). These interpretations align with the model in which many resistance substitutions shift the conformational equilibrium of EF-G toward states with reduced fusidic acid affinity rather than abolishing binding outright [6], which also accords with the measurable fitness cost we observed. Because no *S. haemolyticus* EF-G structure is yet available, these structural inferences are based on homology to *S. aureus* EF-G; however, the complete conservation of the relevant residues makes the inference robust.

Phenotypically, EF-G (FusA) mutants exhibited a marked growth defect: mean doubling time increased by approximately 15 min relative to wild-type parental strains (Cohen’s *d* ≈ 2.0). We used Welch’s *t* test because of unequal group sizes and heterogeneity of variance; a nonparametric Mann–Whitney *U* test produced concordant results, supporting the robustness of the finding. The 95% confidence interval for the mean difference (9.71–20.23 min) indicates that the effect is both statistically significant and biologically meaningful.

Because EF-G is essential for translation, non-synonymous *fusA* mutations are expected to impose fitness costs. In *S. aureus*, such mutations have been associated with reduced fitness and broad metabolic reprogramming, including changes in central carbon metabolism, nucleotide metabolism, and amino acid biosynthesis; compensatory mutations have been reported that mitigate these costs and stabilize resistant clones in populations [15,31]. Reported compensatory loci in *S. aureus* include positions 16, 66, 67, 70, 71, 284, 287, 376, 416, and 475 [15,32]; notably, residue 71 differs between *S. aureus* and *S. haemolyticus*. In contrast to these reports, we did not detect compensatory mutations in EF-G (FusA) in *S. haemolyticus* after serial propagation to the tenth generation. This absence may reflect species-specific differences in compensatory pathways, the limited number of serial passages performed, or the selective conditions used. Moreover, because resistance was characterized by targeted Sanger sequencing of *fusA* rather than whole-genome sequencing, we cannot exclude that secondary mutations outside *fusA* contributed to the observed MIC levels or fitness, or that compensatory changes elsewhere in the genome occurred but were not detected; in *S. aureus*, such compensatory and fitness-modifying changes have been reported both within *fusA* and in genes affecting central carbon, nucleotide, and amino-acid metabolism [15,31]. Whole-genome sequencing of parental and resistant isolates at multiple time points is therefore warranted to fully resolve the resistance and compensatory landscape.

Among the sequence types examined, isolates of ST3 produced a larger number of induced variants (n = 23) and exhibited a broader mutation spectrum (n = 8 distinct substitutions) than isolates of ST1, ST29, and ST42. However, because the study included a limited number of strains and sequence types, these observations are preliminary and do not permit definitive conclusions about differences in in vitro inducibility among various sequence types.

Outside *S. aureus*, EF-G (FusA) mutations have been reported in FA-resistant *S. epidermidis* and *S. pseudintermedius* [11,26,29,33]. For example, clinical *S. epidermidis* isolates have been reported with L461K without accompanying compensatory changes [26], and V599I was reported in isolates from pork together with plasmid-borne *fusB* [33]. In *S. pseudintermedius* from canine sources, EF-G (FusA) substitutions included single-locus variants (V90I, I461T), double mutations (A376V, P404L), and combinations of *fusC* with EF-G changes (I61Y, T62S) [29]. Collectively, these data suggest that the propensity for compensatory evolution after EF-G alteration varies among staphylococcal species and ecological contexts.

This study has several limitations. First, the EF-G (FusA) substitutions reported here were generated by in vitro selection rather than being recovered from clinical *S. haemolyticus* isolates; therefore, their prevalence and clinical relevance remain to be established. In our prior surveillance, FA resistance in clinical *S. haemolyticus* was predominantly mediated by *fusB* or *fusC* [13]; systematic screening of clinical collections for EF-G (FusA) mutations is warranted. Second, the wild-type comparator group for doubling time analysis was small (n = 5), which limits precision and statistical power; although nonparametric testing supported our conclusions, additional biological replicates and independent strain backgrounds would strengthen inference. Third, compensatory mutations in *fusA* may require more generations or different selective regimes than those applied here; longer-term evolution experiments with whole-genome sequencing at multiple time points would better resolve the dynamics of compensation and identify additional loci that contribute to resistance or fitness effects. Fourth, only five parental isolates were studied, and most resistant mutants arose from two ST3 strains (SH53 and SH54); strain-specific factors—such as baseline growth rate, intrinsic mutation rate (e.g., hypermutator phenotypes), and lineage background—may influence the frequency and spectrum of *fusA* mutations, so the predominance of ST3-derived mutants may reflect strain-specific mutability rather than a true lineage difference in resistance potential. Reassuringly, resistant mutants were nonetheless recovered from all five sequence types (ST3, ST42, ST1, and ST29), indicating that the capacity for *fusA*-mediated resistance is shared across lineages; larger, lineage-balanced panels with formal mutation-rate measurement (e.g., fluctuation analysis) will be required to resolve possible lineage-specific effects.

## 4. Materials and Methods

### 4.1. Bacterial Strains

Five representative *S. haemolyticus* isolates that were susceptible to fusidic acid (FA) and belonged to distinct sequence types were selected from previous studies [13,19,22]. The isolates comprised two ST3 strains (SH53, SH54), one ST1 strain (SH141), one ST29 strain (SH29), and one ST42 strain (SH43). Susceptibility to FA was defined according to EUCAST criteria, with MIC ≤ 1 µg/mL considered susceptible [5]. MIC determination was repeated at least three times independently for all isolates.

### 4.2. In Vitro Induction of Fusidic Acid Resistance

The in vitro induction protocol was adapted from previously published methods [16]. Briefly, FA-susceptible isolates were grown overnight in Mueller–Hinton broth (MHB) [BD Difco, Sparks, MD, USA] at 37 °C. Cultures were adjusted to an optical density at 600 nm (OD_600_) of 0.30–0.40 and plated onto Mueller–Hinton agar (MHA) [BD Difco, Sparks, MD, USA] supplemented with 4 µg/mL fusidic acid [Sigma-Aldrich (Merck), St. Louis, MO, USA]. Plates were incubated at 37 °C for 48 h. All visible colonies that grew on FA-containing agar were collected without any preselection and stored for subsequent *fusA* mutation analysis; every collected colony was individually subjected to *fusA* amplification and Sanger sequencing. To evaluate the stability of induced resistance, selected resistant colonies were serially passaged for ten generations in MHB [BD Difco, Sparks, MD, USA] at 37 °C without antibiotic selection; mutational profiles after passaging were compared with those of the parental strains.

### 4.3. fusA Mutation Analysis

The *fusA* was amplified by polymerase chain reaction (PCR) and analyzed by Sanger sequencing. The primer sequences were FusA-F1 5′-CTGAGTGTGTTCCGTCAC-3′ and FusA-R1 5′-CTCTCATGATAGTTTCTCACC-3′. PCR conditions were as follows: initial denaturation at 98 °C for 5 min; 35 cycles of 98 °C for 30 s, 53 °C for 30 s, and 72 °C for 50 s; and a final extension at 72 °C for 5 min. The expected amplicon size was 2348 bp. Sanger sequencing was performed using primers FusA-F1, FusA-R1, FusA-F2 (5′-AATAAGATGGCTCATGCTTAG-3′) and FusA-R2 (5′-CAGATGAATCGACATCATGG-3′). Sequence reads were aligned to reference *fusA* sequences from *S. haemolyticus* strain ATCC 29970 (GenBank accession CP035291.1) and *S. haemolyticus* ST3 strain VB19458 (GenBank accession CP045187.2), as described previously [22]. Comparative analyses including the *S. aureus fusA* sequence (GenBank accession AJ237696.1) were performed to identify conserved and divergent loci, as reported earlier [15].

### 4.4. Growth Assay and Doubling Time Determination

The growth assay was adapted from published protocols [15,34,35]. A single colony of each strain was inoculated into 3 mL tryptic soy broth (TSB) and incubated overnight at 37 °C with shaking. The following day, 0.1 mL of the overnight culture was transferred into 0.9 mL fresh TSB and the suspension was adjusted to OD_600_ = 0.5. From this standardized inoculum, serial ten-fold dilutions (10^−3^ to 10^−7^) were prepared; 0.1 mL of each dilution was spread onto TSB agar plates, which were incubated overnight at 37 °C for colony-forming unit (CFU) enumeration.

In parallel, at least 1 mL of the OD_600_ = 0.5 inoculum was incubated in TSB at 37 °C for 6 h under identical conditions. After 6 h, cultures were serially diluted (10^−3^ to 10^−7^) and plated as described above. CFU counts were used to calculate the number of generations (n) and the doubling time (DT). The number of generations was calculated as

$$n=\frac{{log}_{10}N_{t}-{log}_{10}N_{0}}{{log}_{10}2}$$

where *N*_0_ is the CFU at time 0 (OD_600_ = 0.5) and *Nₜ* is the CFU after 6 h. Doubling time was calculated as DT = t/n, where t is the incubation time in hours (6 h).

### 4.5. Statistical Analysis

Descriptive data are presented as mean ± standard deviation (SD). Because group sample sizes and variances were unequal, comparisons of mean doubling times between wild-type and *fusA* mutant isolates were performed using Welch’s two-sample *t* test (two-tailed). To assess robustness against departures from normality, a Mann–Whitney *U* test (Wilcoxon rank-sum test) was used as a nonparametric alternative. Effect size was reported as Cohen’s *d* calculated without assuming equal pooled variances, using the following formula; 95% confidence intervals for mean differences were also provided.

$$d=\frac{{\bar{X}}_{1}-{\bar{X}}_{2}}{\sqrt{\frac{{SD}_{1}^{2}+{SD}_{2}^{2}}{2}}}$$

All statistical analyses were conducted using the Statistical Package for the Social Sciences (SPSS) for Windows, version 17.0 (Chicago, IL, USA). A *p*-value of ≤0.05 was considered indicative of statistical significance.

## 5. Conclusions

In summary, EF-G (FusA) substitutions can be rapidly selected in vitro in *S. haemolyticus* and confer a range of FA-resistance phenotypes, including both previously described substitutions and novel mutations (L430S, E433G, H457N, H457L, A655G). These mutations were associated with increased doubling times and, under our experimental conditions (ten serial passages without antibiotic selection, with re-sequencing of *fusA*), did not give rise to detectable compensatory mutations within *fusA*; however, compensatory evolution may require longer-term propagation, larger populations, or whole-genome analysis, and cannot be excluded under other conditions. Given the clinical importance of *S. haemolyticus*, further work is needed to determine the frequency of *fusA*-mediated resistance in clinical isolates, to characterize the structural and mechanistic basis of novel substitutions, and to assess the potential for compensatory evolution under clinically relevant selective pressures.

## Figures and Tables

**Table 1:** Amino acid and corresponding codon differences in EF-G (FusA) between *S. aureus* and *S. haemolyticus*.

Residue	*S. aureus* (AJ237696.1)		*S. haemolyticus* (CP045187.2) ^a,b^	
	Amino Acid	Codon	Amino Acid	Codon
4	Glu (E)	GAA	Asp (D)	GAC
8	Glu (E)	GAA	Lys (K)	AAA
9	Lys (K)	AAA	Asn (N)	AAC
71	Ala (A)	GCT	Gln (Q)	CAA
73	Glu (E)	GAA	Asp (D)	GAT
145	Glu (E)	GAA	Asp (D)	GAC
156	Gln (Q)	CAA	Asp (D)	GAT
195	Glu (E)	GAA	Asp (D)	GAC
204	Leu (L)	TTA	Lys (K)	AAA
205	Asp (D)	GAT	Glu (E)	GAA
212	Ala (A)	GCT	Ser (S)	TCT
213	Ser (S)	AGC	Asn (N)	AAT
222	Ser (S)	AGC	Asn (N)	AAC
238	Ser (S)	TCT	Ala (A)	GCA
242	Glu (E)	GAA	Asn (N)	AAT
250	Asn (N)	AAC	Asp (D)	GAC
272	Asp (D)	GAC	Asn (N)	AAC
288	Ile (I)	ATT	Val (V)	GTA
293	Ser (S)	AGC	Glu (E)	GAA
303	Ala (A)	GCA	Pro (P)	CCA
335	Met (M)	ATG	Leu (L)	TTA
341	Val (V)	GTT	Ile (I)	ATT
347	Gly (G)	GGT	Asp (D)	GAC
488	Ser (S)	TCA	Gln (Q)	CAA
527	Ala (A)	GCT	Ser (S)	TCT
567	Tyr (Y)	TAT	Phe (F)	TTT
625	Ser (S)	TCT	Ala (A)	GCT
684	Glu (E)	GAA	Asp (D)	GAT

^a^ The EF-G (FusA) sequences of the five selected fusidic acid (FA)-susceptible *S. haemolyticus* isolates are identical to that of the *S. haemolyticus* reference strain VB19458 (GenBank accession no. CP045187.2). ^b^ *S. haemolyticus* strain VB19458 (CP045187.2) carries two synonymous nucleotide substitutions at codons 176 (ATC → ATT) and 342 (AAG → AAA) relative to ATCC 29970 (CP035291.1), which do not alter the encoded amino acids.

**Table 2:** Genetic alterations in *fusA*, isolate numbers, and FA MICs of in vitro-induced FA-resistant strains of selected *S. haemolyticus* isolates.

Amino Acid Changes in EF-G (FusA)	Isolates (No.)	FA MICs (µg/mL)	Mutations of *fusA* ^a^
Wild Type	5	<=1	N/A
Q115L	3	64	A344T (CAA → CTA)
L430S	1	16	T1289C (TTA → TCA)
E433G	1	16	A1298G (GAA → GGA)
G452C	2 ^c^	32	G1354T (GGT → TGT)
H457Y	14 ^b^	64–128	C1369T (CAC → TAC)
H457N	1	>256	C1369A (CAC → AAC)
H457L	1	128	A1370T (CAC → CTC)
H457Q	2	16	C1371A (CAC → CAA)
R464L	2	16	G1391T (CGT → CTT)
A655G	1	32	C1964G (GCA → GGA)
Silent Mutations of EF-G (FusA)			
I176I	10 ^b^	N/A	C528T (ATC → ATT)
G386G	10 ^b^	N/A	T1158C (GGT → GGC)
G451G	1 ^c^	N/A	T1353A (GGT → GGA)

^a^ The mutations of the 1st, 5th, and 10th generations of each in vitro-induced FA-resistant isolate were identical. ^b^ There were 10 isolates carrying H457Y and two silent mutations (I176I and G386G) of EF-G (FusA). ^c^ There was one isolate carrying G452C and one silent mutation (G451G) of EF-G (FusA).

**Table 3:** Doubling times (DT, min) of parental wild-type strains and EF-G (FusA) mutant isolates. Each doubling time measurement for an individual strain represents the mean of three independent biological replicates. For mutations present in multiple independent strains, the presented value is the grand mean of the doubling times across all strains carrying that mutation; per-strain values are detailed in the footnotes. Statistical comparisons of DT between wild-type and mutant strains were performed as described in the text.

Strain (ST)		Amino Acid Changes in EF-G (FusA)									
	WT	Q115L	L430S	E433G	G452C	H457Y	H457N	H457L	H457Q	R464L	A655G
SH53(ST3)	43.1	58.4 ^a^	63.1	57.0	60.8 ^b^	54.7	-	-	60.6 ^c^	48.9 ^d^	52.3
SH54(ST3)	41.9	-	-	-	-	54.7 ^e^	-	-	-	-	-
SH43(ST42)	40.2	-	-	-	-	47.6	-	41.7	-	-	-
SH141(ST1)	34.0	-	-	-	-	63.2 ^f^	-	-	-	-	-
SH29(ST29)	44.7	-	-	-	-	-	56.1	-	-	-	-
**Comparison of the DT between WT isolates (n = 5) and EF-G (FusA) mutant isolates (n = 28)**											
**Test**			**Statistic**	**df**		***p* value**		**Effect size**		**95% CI (difference)**	
Welch two-sample t			t = −6.34	≈10.0		<0.0001 *		Cohen’s d = 2.02		9.71 to 20.23 min	
Mann–Whitney U			U = 6.5	-		≈0.0015 *		-		-	

^a^ n = 3 strains (individual mean DTs: 58.7, 67.8, 48.8 min); ^b^ n = 2 strains (individual mean DTs: 57.7, 63.8 min); ^c^ n = 2 strains (individual mean DTs: 62.1, 59.0 min); ^d^ n = 2 strains (individual mean DTs: 44.1, 53.6 min); ^e^ n = 10 strains (individual mean DTs: 43.1, 62.0, 56.3, 49.6, 44.3, 60.6, 66.1, 50.4, 47.6, 66.5 min); ^f^ n = 2 strains (individual mean DTs: 65.9, 60.4 min); * *p* <= 0.05.

## Data Availability

The data supporting the findings of this study are available within the article and its Supplementary Materials. The reference EF-G (fusA) sequences analyzed are available in GenBank under accession numbers AJ237696.1, CP045187.2 (VB19458), and CP035291.1 (ATCC 29970).

## Supplementary Materials

The following supporting information can be downloaded at https://www.mdpi.com/article/10.3390/antibiotics15060589/s1. Figure S1-1: Comparison of EF-G (FusA) amino acid sequences (total 693 amino acids) of *S. aureus* (GenBank: AJ237696.1) and *S. haemolyticus* strain VB19458 (GenBank: CP045187.2). Amino acids highlighted in bold and shaded in gray indicate the inconsistent loci. Figure S1-2: Comparison of *fusA* sequences of *S. aureus* (GenBank: AJ237696.1) and *S. haemolyticus* strain VB19458 (GenBank: CP045187.2). Codons of different amino acids are presented in bold and underlined; distinct gene sequences are shaded in gray.

## Abbreviations

The following abbreviations are used in this manuscript:

FA	fusidic acid
EF-G	elongation factor-G
MSSA	methicillin-susceptible *Staphylococcus aureus*
MRSA	methicillin-resistant *Staphylococcus aureus*
CLSI	Clinical and Laboratory Standards Institute
EUCAST	European Committee on Antimicrobial Susceptibility Testing
MIC	minimal inhibitory concentration
ST	sequence type
MHB	Mueller–Hinton broth
OD	optical density
MHA	Mueller–Hinton agar
PCR	polymerase chain reaction
TSB	tryptic soy broth
CFU	colony forming unit
DT	doubling time
SD	standard deviation
SPSS	Statistical Package for the Social Sciences
