# Supplementary material for: Elongation Factor-G (fusA) Mutations That Confer Fusidic Acid Resistance in Staphylococcus haemolyticus
Source: Antibiotics (Basel). 2026 Jun 9;15(6):589. doi: 10.3390/antibiotics15060589 (PMC13295663; doi:10.3390/antibiotics15060589)
Supplement: Supplementary file 1 [file antibiotics-15-00589-s001.zip › antibiotics-4349255-supplementary.pdf]

Figure S1-1. The comparison of EF-G (FusA) amino acid sequences (total 693 amino acids) of *S. aureus* (GenBank: AJ237696.1, upper lane) and *S. haemolyticus* strain (GenBank: VB19458/CP045187, lower lane). The amino acids highlighted in bold and shaded in gray indicate the inconsistent locus.

MAR**E**FSL**EK**TRNIGIMAHIDAGKTTTTERILYYTGRIHKIGETHEGASQMDWMEQEQDRG
MAR**D**FSL**KN**TRNIGIMAHIDAGKTTTTERILYYTGRIHKIGETHEGASQMDWMEQEQDRG

ITITSAATTA**A**W**E**GHRVNIIDTPGHVDFTVEVERSLRVLDGAVTVLDAQSGVEPQTETVW
ITITSAATTA**Q**W**D**GHRVNIIDTPGHVDFTVEVERSLRVLDGAVTVLDAQSGVEPQTETVW

RQATTYGVPRIVFVNKMDKLGANF**E**YSVSTLHDRL**Q**ANAAPIQLPIGAEDEFEAIIDLVE
RQATTYGVPRIVFVNKMDKLGANF**D**YSVSTLHDRL**D**ANAAPIQLPIGAEDEFEAIIDLVE

MKCFKYTNDLGTEI**E**EIEIPEDH**LD**RAEEAR**AS**LIEAVAET**S**DELMEKYLGDEEISV**S**EL
MKCFKYTNDLGTEI**D**EIEIPEDH**KE**RAEEAR**SN**LIEAVAET**N**DELMEKYLGDEEISV**A**EL

K**E**AIRQATT**N**VEFYPVLCGTAFKNKGVQLML**D**AVIDYLPSPLDVKPI**I**GHRA**S**NPEEEVI
K**N**AIRQATT**D**VEFYPVLCGTAFKNKGVQLML**N**AVIDYLPSPLDVKPI**V**GHRA**E**NPEEEVI

AK**A**DDSAEFAALAFKVMTDPYVGKLTFFRVYSGT**M**TSGSY**V**KNSTK**G**KRERVGRLLQMHA
AK**P**DDSAEFAALAFKVMTDPYVGKLTFFRVYSGT**L**TSGSY**I**KNSTK**D**KRERVGRLLQMHA

NSRQEIDTVYSGDIAAAVGLKDTGTGDTLCGEKNDIILESMEFPEPVIHLSVEPKSKADQ
NSRQEIDTVYSGDIAAAVGLKDTGTGDTLCGEKNDIILESMEFPEPVIHLSVEPKSKADQ

DKMTQALVKLQEEDPTFHAHTDEETGQVIIGGMGELHLDILVDRMKKEFNVECNVGAPMV
DKMTQALVKLQEEDPTFHAHTDEETGQVIIGGMGELHLDILVDRMKKEFNVECNVGAPMV

SYRETFK**S**SAQVQGKFSRQSGGRGQYGDVHIEFTPNETGAGFEFEN**A**IVGGVVPREYIPS
SYRETFK**Q**SAQVQGKFSRQSGGRGQYGDVHIEFTPNETGAGFEFEN**S**IVGGVVPREYIPS

VEAGLKDAMENGVLAGYPLIDVKAKL**Y**DGSYHDVDSSEMAFKIAASLALKEAAKKCDPVI
VEAGLKDAMENGVLAGYPLIDVKAKL**F**DGSYHDVDSSEMAFKIAASLALKEAAKKCDPVI

LEPMMKVTIEMPEEYMGDIMGDVT**S**RRGRVDGMEPRGNAQVVNAYVPLSEMFGYATSLRS
LEPMMKVTIEMPEEYMGDIMGDVT**A**RRGRVDGMEPRGNAQVVNAYVPLSEMFGYATSLRS

NTQGRGTYTMYFDHYAEVPKSIA**E**DIIKKNKGE
NTQGRGTYTMYFDHYAEVPKSIA**D**DIIKKNKGE

Figure S1-2. The comparison of *fusA* sequences of *S. aureus* (GenBank: AJ237696.1, upper lane) and *S. haemolyticus* strain (GenBank: VB19458/CP045187, lower lane). Codons of different amino acids between *S. aureus* and *S. haemolyticus* are presented in bold and underlined, whereas distinct gene sequences are shaded in gray.

ATGGCTAGA**GAA**TTTTCATTA**GAAAAA**ACTCGTAATATCGGTATCATGGCTCACATTGAT
ATGGCTAGA**GAC**TTTTCTTTG**AAAAAC**ACTCGTAACATCGGTATCATGGCTCACATTGAT

GCTGGTAAAACGACTACGACTGAACGTATTCTTTATTACACTGGCCGTATCCACAAAATT
GCTGGTAAAACAACTACGACTGAACGTATTCTTTACTATACAGGTCGTATCCACAAAATT

GGTGAAACACACGAAGGTGCTTCACAAATGGACTGGATGGAGCAAGAACAAGACCGTGGT
GGTGAAACACACGAAGGTGCTTCACAAATGGACTGGATGGAACAAGAACAAGACCGTGGT

ATTACTATCACATCTGCTGCAACAACAGCA**GCT**TGG**GAA**GGTCACCGTGTAAACATTATC
ATTACAATCACATCAGCTGCTACAACAGCT**CAA**TGG**GAT**GGCCACCGTGTAAACATTATC

GATACACCTGGACACGTAGACTTCACTGTAGAAGTTGAACGTTCATTACGTGTACTTGAC
GATACTCCAGGACACGTAGACTTCACTGTTGAGGTTGAACGTTCTTTACGTGTACTTGAT

GGAGCAGTTACAGTACTTGATGCACAATCAGGTGTTGAACCACAAACTGAAACAGTTTGG
GGTGCGGTTACTGTACTTGATGCTCAATCAGGTGTAGAACCTCAAACTGAAACGGTTTGG

CGTCAGGCTACAACTTATGGTGTTCCACGTATCGTATTTGTAAACAAAATGGACAAATTA
CGTCAAGCTACAACTTACGGTGTACCTCGTATCGTATTCGTAAACAAAATGGATAAATTA

GGTGCTAACTTC**GAA**TACTCTGTAAGTACATTACATGATCGTTTA**CAA**GCTAACGCTGCT
GGCGCTAACTTC**GAC**TATTCTGTAAGTACATTACATGATCGTTTA**GAT**GCTAACGCTGCA

CCAATCCAATTACCAATTGGTGCGGAAGACGAATTCGAAGCAATCATTGACTTAGTTGAA
CCTATCCAATTACCAATTGGTGCCGAAGACGAATTTGAAGCTATCATCGACTTAGTAGAA

ATGAAATGTTTCAAATATACAAATGATTTAGGTACTGAAATT**GAA**GAAATTGAAATTCCT
ATGAAATGCTTCAAATATACTAATGACTTAGGTACTGAAATT**GAC**GAAATTGAAATCCCT

GAAGACCAC**TTAGAT**AGAGCTGAAGAAGCTCGT**GCTAGC**TTAATCGAAGCAGTTGCAGAA
GAAGATCAC**AAAGAA**AGAGCAGAAGAAGCTCGT**TCTAAT**TTAATCGAAGCAGTTGCAGAA

ACT**AGC**GACGAATTAATGGAAAAATATCTTGGTGACGAAGAAATTTCAGTT**TCT**GAATTA
ACT**AAC**GATGAATTAATGGAAAAATATCTTGGTGATGAAGAAATTTCAGTA**GCA**GAATTA

AAA**GAA**GCTATCCGCCAAGCTACTACT**AAC**GTAGAATTCTACCCAGTACTTTGTGGTACA

AAA**AAT**GCTATTCGTCAAGCAACTACT**GAC**GTAGAATTCTACCCAGTACTTTGCGGTACA

GCTTTCAAAAACAAAGGTGTTCAATTAATGCTT**GAC**GCTGTAATTGATTACTTACCTTCA
GCATTCAAAAACAAAGGTGTTCAATTAATGCTG**AAC**GCAGTAATTGACTACTTACCATCA

CCACTAGACGTTAAACCAATT**ATT**GGTCACCGTGCT**AGC**AACCCTGAAGAAGAAGTAATC
CCTTTAGATGTTAAACCAATC**GTA**GGCCATCGTGCT**GAA**AATCCTGAAGAAGAAGTAATT

GCGAAA**GCA**GACGATTCAGCTGAATTCGCTGCATTAGCGTTCAAAGTTATGACTGACCCT
GCTAAA**CCA**GACGATTCAGCTGAATTCGCTGCATTAGCATTCAAAGTTATGACTGACCCT

TATGTTGGTAAATTAACATTCTTCCGTGTGTATTCAGGTACA**ATG**ACATCTGGTTCATAC
TATGTTGGTAAATTAACTTTCTTCCGTGTATATTCAGGTACT**TTA**ACATCAGGTTCTTAC

GTTAAGAACTCTACTAAA**GGT**AAACGTGAACGTGTAGGTCGTTTATTACAAATGCACGCT
ATTAAGAACTCAACTAAA**GAC**AAACGTGAACGTGTAGGTCGTTTATTACAAATGCACGCT

AACTCACGTCAAGAAATCGATACTGTATACTCTGGAGATATCGCGGCTGCGGTAGGTCTT
AACTCACGTCAAGAAATCGACACTGTTTACTCAGGCGATATCGCTGCTGCAGTTGGTCTT

AAAGATACAGGTACTGGTGATACTTTATGTGGTGAGAAAAATGACATTATCTTGGAATCA
AAAGATACAGGTACTGGTGACACATTATGTGGTGAAAAGAATGACATTATCTTAGAATCA

ATGGAATTCCCAGAGCCAGTTATTCACTTATCAGTAGAGCCAAAATCTAAAGCTGACCAA
ATGGAATTCCCTGAACCAGTTATCCACTTATCAGTAGAACCAAAATCTAAAGCTGACCAA

GATAAAATGACTCAAGCTTTAGTTAAATTACAAGAAGAAGACCCAACATTCCATGCACAC
GATAAAATGACTCAAGCTTTAGTTAAATTACAAGAAGAAGACCCTACTTTCCATGCACAC

ACTGACGAAGAAACTGGACAAGTTATCATCGGTGGTATGGGTGAGCTTCACTTAGACATC
ACAGATGAAGAAACTGGACAAGTTATCATCGGTGGTATGGGTGAGTTACACTTAGACATC

TTAGTAGACCGTATGAAGAAAGAATTCAACGTTGAATGTAACGTAGGTGCTCCAATGGTT
TTAGTTGACCGTATGAAGAAAGAATTCAACGTTGAATGTAATGTAGGTGCCCCAATGGTT

TCATATCGTGAAACATTCAAA**TCA**TCTGCACAAGTTCAAGGTAAATTCTCTCGTCAATCT
TCATATCGTGAAACGTTCAAG**CAA**TCTGCACAAGTTCAAGGTAAATTCTCTCGTCAATCT

GGTGGTCGTGGTCAATACGGTGATGTTCACATTGAATTCACACCAAACGAAACAGGCGCA
GGTGGTCGTGGTCAATACGGTGATGTTCACATTGAATTCACACCAAACGAAACTGGCGCA

GGTTTCGAATTCGAAAAC**GCT**ATCGTTGGTGGTGTAGTTCCTCGTGAATACATTCCATCA
GGTTTCGAATTCGAAAAC**TCT**ATCGTTGGTGGTGTAGTTCCTCGTGAATACATTCCATCA

GTAGAAGCTGGTCTTAAAGATGCTATGGAAAATGGTGTCTTAGCAGGTTATCCTTTAATT
GTTGAAGCTGGTCTTAAAGATGCTATGGAAAACGGTGTATTAGCTGGTTATCCATTAATC

GATGTTAAAGCTAAATTA**TAT**GATGGTTCATACCATGATGTCGATTCATCTGAAATGGCC
GATGTTAAAGCAAAATTA**TTT**GATGGTTCATACCATGATGTCGATTCATCTGAAATGGCC

TTCAAAATTGCTGCATCATTAGCACTTAAAGAAGCTGCTAAAAAATGTGATCCTGTAATC
TTCAAAATTGCTGCATCATTAGCACTTAAAGAAGCTGCTAAAAAATGTGATCCAGTTATC

TTAGAACCAATGATGAAAGTAACTATTGAAATGCCTGAAGAGTACATGGGTGATATCATG
TTAGAACCAATGATGAAAGTAACAATCGAAATGCCTGAAGAATACATGGGTGATATCATG

GGTGACGTAACA**TCT**CGTCGTGGACGTGTTGATGGTATGGAACCTCGTGGTAATGCACAA
GGTGACGTAACA**GCT**CGTCGTGGACGTGTTGATGGTATGGAACCTCGTGGTAATGCACAA

GTTGTTAATGCTTATGTACCACTTTCAGAAATGTTCGGTTATGCAACATCATTACGTTCA
GTTGTTAACGCTTATGTACCACTTTCAGAAATGTTCGGTTACGCAACTTCATTACGTTCT

AACACTCAAGGTCGCGGTACTTACACTATGTACTTCGATCACTATGCTGAAGTTCCAAAA
AACACTCAAGGTCGCGGTACTTACACTATGTACTTCGATCACTATGCAGAAGTTCCAAAA

TCAATCGCT**GAA**GATATTATCAAGAAAAATAAAGGTGAATAA
TCAATTGCT**GAT**GATATCATCAAAAAAAATAAAGGTGAATAA
